# Supplementary material for: The AKT2/SIRT5/TFEB pathway as a potential therapeutic target in non-neovascular AMD
Source: Nat Commun. 2024 Jul 21;15:6150. doi: 10.1038/s41467-024-50500-z (PMC11271488; doi:10.1038/s41467-024-50500-z)
Supplement: Supplementary file 1 — Supplementary Information [file 41467_2024_50500_MOESM1_ESM.pdf]

Supplementary Information

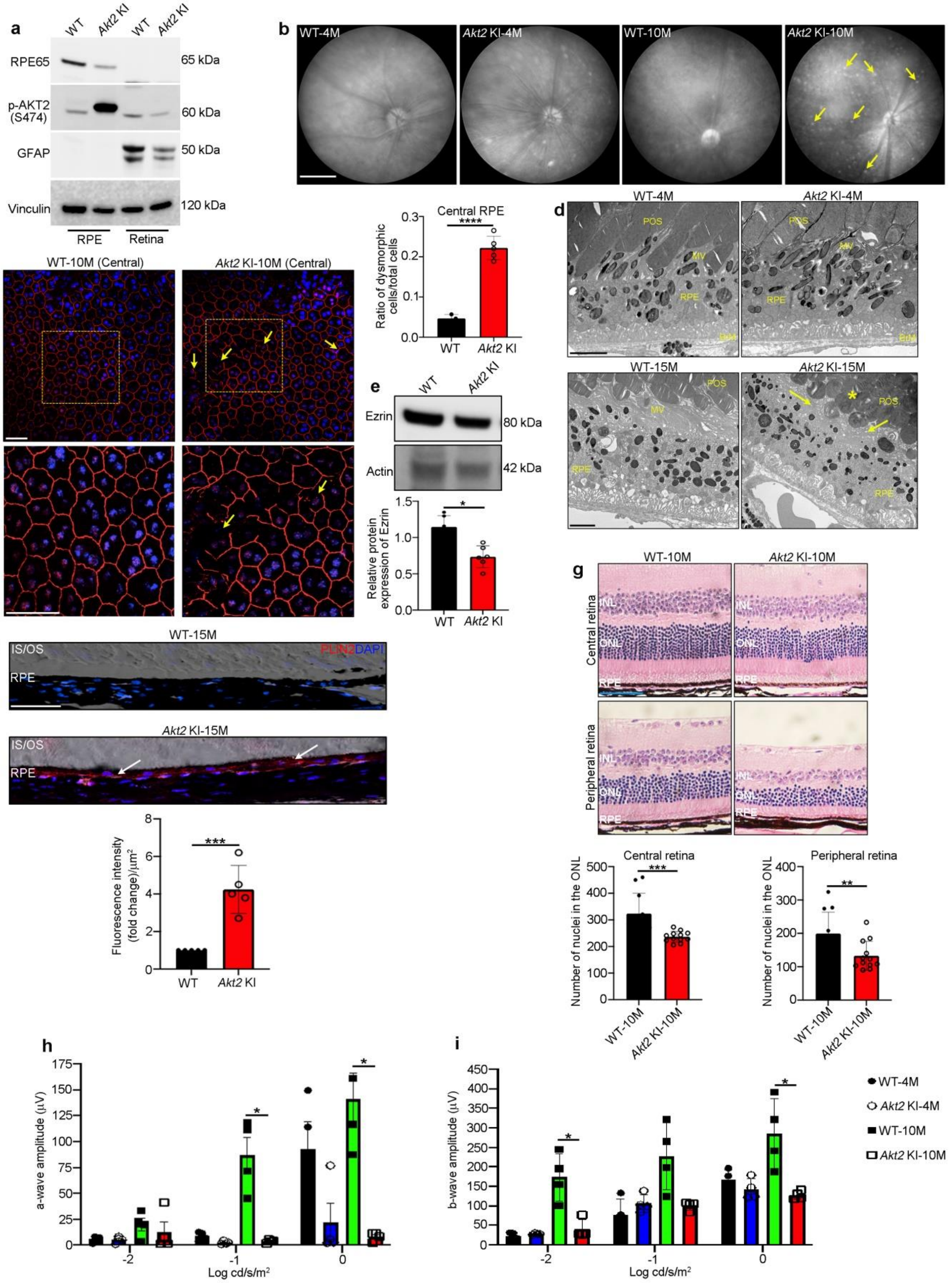

Supplementary Figure 1: ***Akt2 KI mice develop an AMD-like phenotype***. (**a**) Western blot showing specific upregulation of AKT2 in RPE cells and not in the neurosensory retina of *Akt2* KI mice, compared to WT. n=3. (**b**) Fundus photographs showing accumulation of

autofluorescent foci (arrows) in 10-month-old *Akt2* KI mice, but not in age-matched controls or young animals. n=5. (**c**) Immunostaining with ZO-1 on RPE flatmount from 10-month-old *Akt2* KI mice showing alterations in the normal cobblestone-like morphology in the central retinal region, with increase in dysmorphic cell number (arrows), not seen in age-matched WT. n=4. Scale bar= 50 μm (Zoomed Inset= 80 μm). (**d**) Transmission electron micrographs showing loss of microvilli (MV; arrows in *Akt2* KI) and abnormal photoreceptor outer segments (POS; asterisk in *Akt2* KI) in 15-month-old *Akt2* KI RPE cells, but not in age-matched WT or young (4 month old) *Akt2* KI mice. n=5. Scale bar= 2 μm. (**e**) Western blot showing decreased expression of ezrin in 10-month-old *Akt2* KI RPE cells, relative to control (WT). n=4. (**f**) Immunofluorescence studies showing increased accumulation of PLIN2 (red; arrows in *Akt2* KI) in 15-month-old *Akt2* KI RPE, compared to WT. n=5. Scale bar= 50 μm. (**g**) Hematoxylin-eosin stained slides were used to quantify the number of nuclei in the ONL as a measure of photoreceptor degeneration, showing a significant decrease in number of ONL nuclei in 10 month old *Akt2* KI, compared to age-matched WT. n=5. (**h**,**i**) Electroretinography analysis showing decrease in scotopic (**h**) a-wave and (**i**) b-wave amplitudes in 10- month- old *Akt2* KI mice, compared to controls. Such changes were not seen in young (4 month old) mice. n=4. All values are Mean ± S.D. ****P<0.0001, ***P<0.001, **P<0.01, *P<0.05. Statistical test used in (**c**, **e**, **f** and **g**) is Student's t-test and in (**h**) is One-way ANOVA followed by Tukey's post-hoc test. The exact p-values are (**c**) P=0.00008 (*Akt2* KI vs WT); (**e**) P= 0.0312 (*Akt2* KI vs WT); (**g**) P=0.0009 (central), P=0.0047 (peripheral) (*Akt2* KI vs WT); (**h**) P=0.0407 (*Akt2* KI-10m vs WT-10m for -1 Log cd.s/m^2), P=0.0366 (*Akt2* KI-10m vs WT-10m for 0 Log cd.s/m^2); (**i**) P=0.0498 (*Akt2* KI-10m vs WT-10m for -2 Log cd.s/m^2), P=0.0486 (*Akt2* KI-10m vs WT-10m for 0 Log cd.s/m^2). Source Data is provided in the Source Data file.

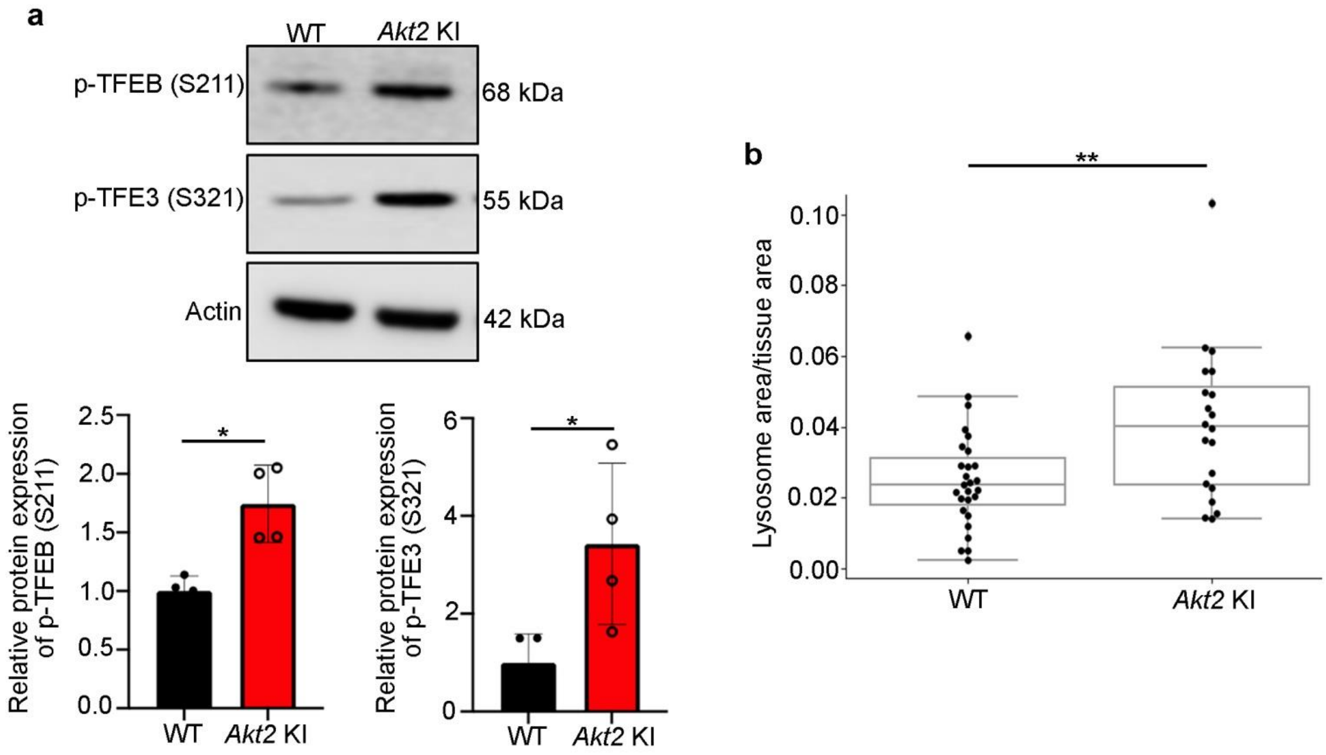

<u>**Supplementary Figure 2**</u>: **Abnormal TFEB/E3 levels and lysosomal characteristics in *Akt2* KI RPE**. (**a**) Western blot showing increased levels of p-TFEB (S211) and p-TFE3 (S321) in RPE cells from 4 month old *Akt2* KI mice, compared to WT, indicating diminished nuclear localization of these transcription factors. n=4. (**b**) Estimation of lysosomal area (normalized to total tissue area) on TEM micrographs from WT and *Akt2* KI RPE showing increase in lysosomal area in *Akt2* KI RPE cells, a characteristic feature of abnormal lysosomes. n= 5 (WT), 4 (*Akt2* KI). **P<0.01, *P<0.05. Statistical test used in (**a** and **b**) is Student's t-test. The exact p-values are (**a**) p-TFEB (S211), P=0.0286 (*Akt2* KI vs WT), p-TFE3 (S321) P=0.0285 (*Akt2* KI vs WT); (**b**) P= 0.0046 (*Akt2* KI vs WT). Source Data is provided in the Source Data file.

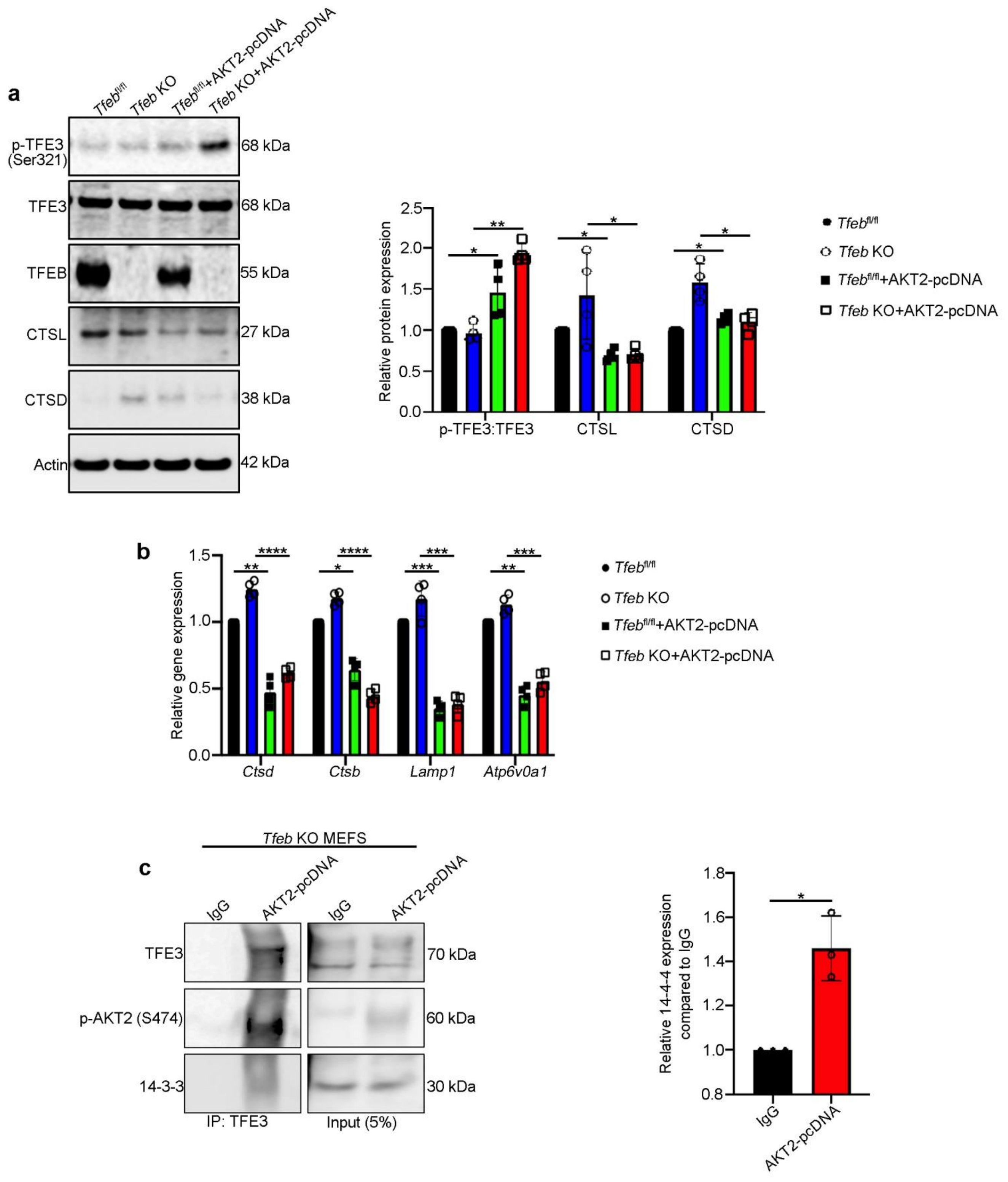

Supplementary Figure 3: ***Akt2 inhibits TFE3-mediated compensatory lysosomal function upon loss of TFEB***. (**a**) Western blot analysis showing that Akt2 overexpression in starved (20 h in serum-free medium) *Tfeb* KO and *Tfeb* floxed mouse embryonic flibroblasts (MEFs),

increased the protein levels of p-TFE3 (S321) and decreased CTSD and CTSL, compared to control (untransfected cells). n=4. (**b**) Expression of *Ctsd*, *Ctsb*, *Lamp1*, and *Atp6v0a1* also showed noticeable downregulation in Akt2 overexpressing and starved *Tfeb* KO and *Tfeb* floxed MEFs compared to controls. n=4. (**c**) Co-immunoprecipitation studies (with pulling down of TFE3 and immunoblotting for 14-3-3) showing that in Akt2 overexpressing *Tfeb* KO MEFs (using AKT2-pcDNA), TFE3 binds to 14-3-3, as compared to IgG pull-down controls from untransfected cells, perhaps accounting for its proteasomal degradation and subsequent inhibition of nuclear translocation. Interestingly, we also found association of p-AKT2 (S474) to the TFE3 pulled down complex (input showing high levels of p-AKT2 due to overexpression), probably signifying binding between the two proteins and subsequent phosphorylation of TFE3 by AKT2. n=4. ****P<0.0001, ***P<0.001, **P<0.01, *P<0.05. Statistical test used in (**a** and **b**) is One-way ANOVA followed by Tukey's post-hoc test and in (**c**) is Student's t-test. The exact p-values are (**a**) p-TFE3:TFE3: P=0.048 (*Tfeb*$^{fl/fl}$+AKT2-pcDNA vs *Tfeb*$^{fl/fl}$), P=0.0098 (*Tfeb* KO+ AKT2-pcDNA vs *Tfeb* KO), CTSL: P=0.0115 (*Tfeb*$^{fl/fl}$+AKT2-pcDNA vs *Tfeb*$^{fl/fl}$), P=0.047 (*Tfeb* KO+ AKT2-pcDNA vs *Tfeb* KO), CTSD: P=0.044 (*Tfeb*$^{fl/fl}$+AKT2-pcDNA vs *Tfeb*$^{fl/fl}$), P=0.048 (*Tfeb* KO+ AKT2-pcDNA vs *Tfeb* KO); (**b**) *Ctsd*: P=0.0059 (*Tfeb*$^{fl/fl}$+AKT2-pcDNA vs *Tfeb*$^{fl/fl}$), P=0.000092 (*Tfeb* KO+ AKT2-pcDNA vs *Tfeb* KO), *Ctsb*: P=0.0104 (*Tfeb*$^{fl/fl}$+AKT2-pcDNA vs *Tfeb*$^{fl/fl}$), P=0.000082 (*Tfeb* KO+ AKT2-pcDNA vs *Tfeb* KO), *Lamp1*: P=0.0006, (*Tfeb*$^{fl/fl}$+AKT2-pcDNA vs *Tfeb*$^{fl/fl}$), P=0.0009 (*Tfeb* KO+ AKT2-pcDNA vs *Tfeb* KO), *Atp6v0a1*: P=0.0018, (*Tfeb*$^{fl/fl}$+AKT2-pcDNA vs *Tfeb*$^{fl/fl}$), P=0.0001 (*Tfeb* KO+ AKT2-pcDNA vs *Tfeb* KO); (**c**) P=0.045 (AKT2-pcDNA vs IgG). Source Data is provided in the Source Data file.

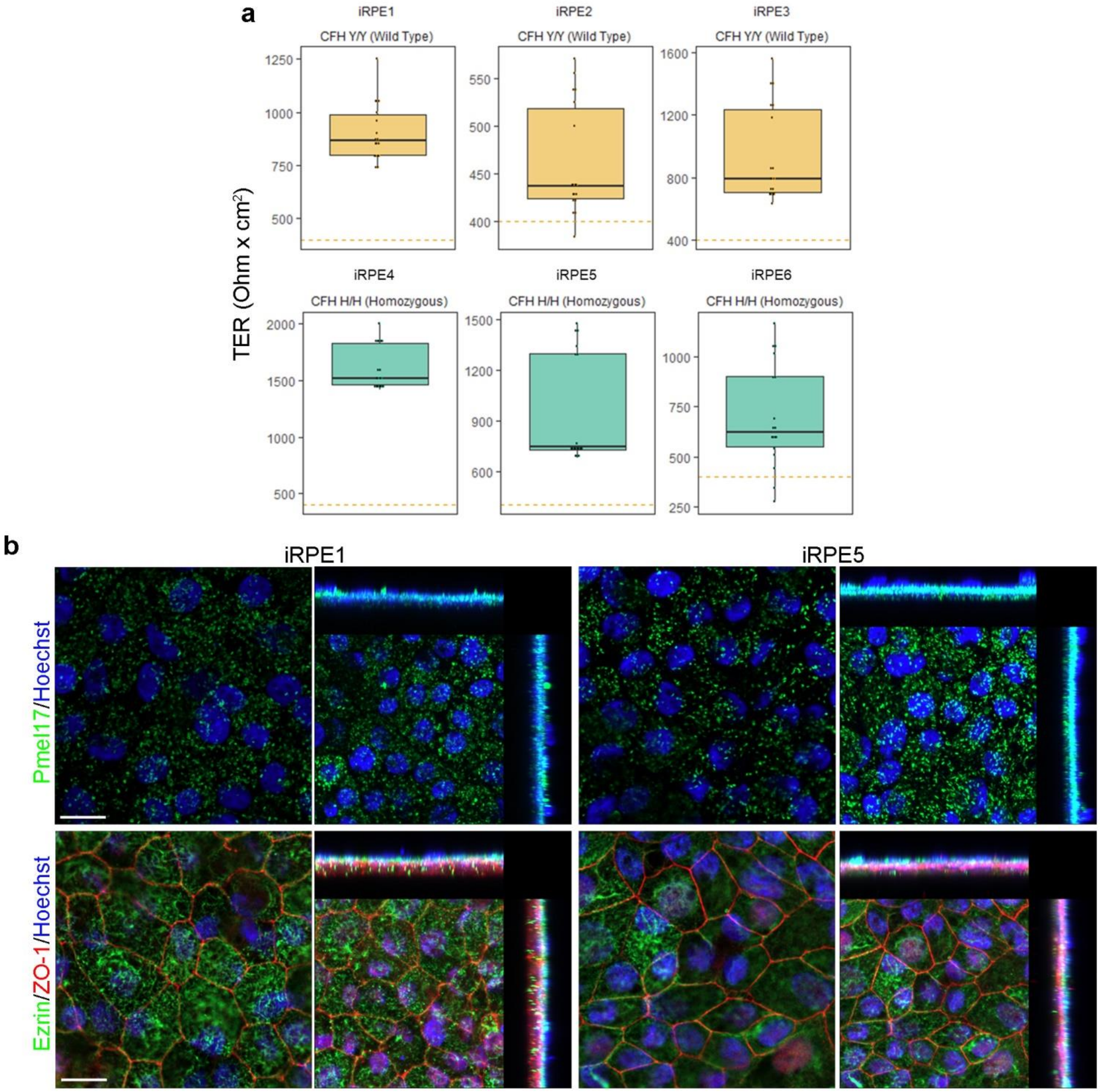

<u>Supplementary Figure 4</u>: iPSC-derived RPE cell characterization. (**a**) TER values showing robust increase in differentiated RPE cells from all the CFH (Y/Y) and CFH (H/H) lines. Dotted line indicated minimum value of TER demonstrated for iPSC-derived RPE cells previously[29]. (**b**) Immunofluorescence studies showing noticeable expression of RPE-specific markers (PMEL17 and Ezrin; Green) along with prevalence of a cobblestone-like morphology (ZO-1;

red) in the iPSC-derived RPE cells from both genotypes. n=3. iRPE1= CFH (Y/Y), iRPE5= CFH (H/H). Source Data is provided in the Source Data file.

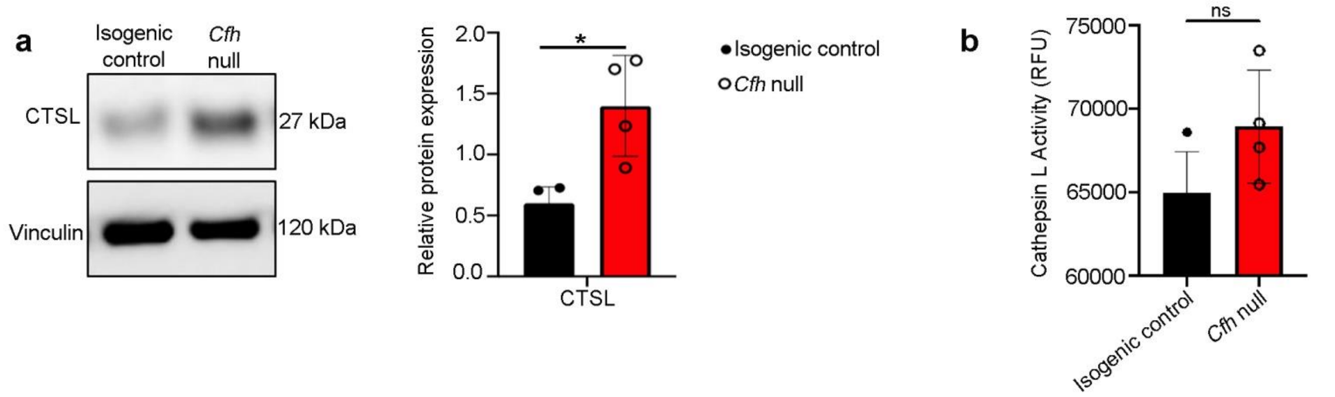

<u>Supplementary Figure 5</u>: ***Loss of CFH does not result in abnormal lysosomal function in iRPE cells***. (**a**) Western blot and densitometric analysis showing significant increase in Cathepsin L (CTSL) protein levels and (**b**) an increase in its activity in *CFH* null ($CFH^{-/-}$) iRPE cells, compared to isogenic controls. n=4. *P<0.05. ns= non-significant. Statistical test used in (**a**) is Student's t-test. The exact p-values are (**a**) P=0.0436 and (**b**) P=01058 (*Cfh* null vs Isogenic control). Source Data is provided in the Source Data file.

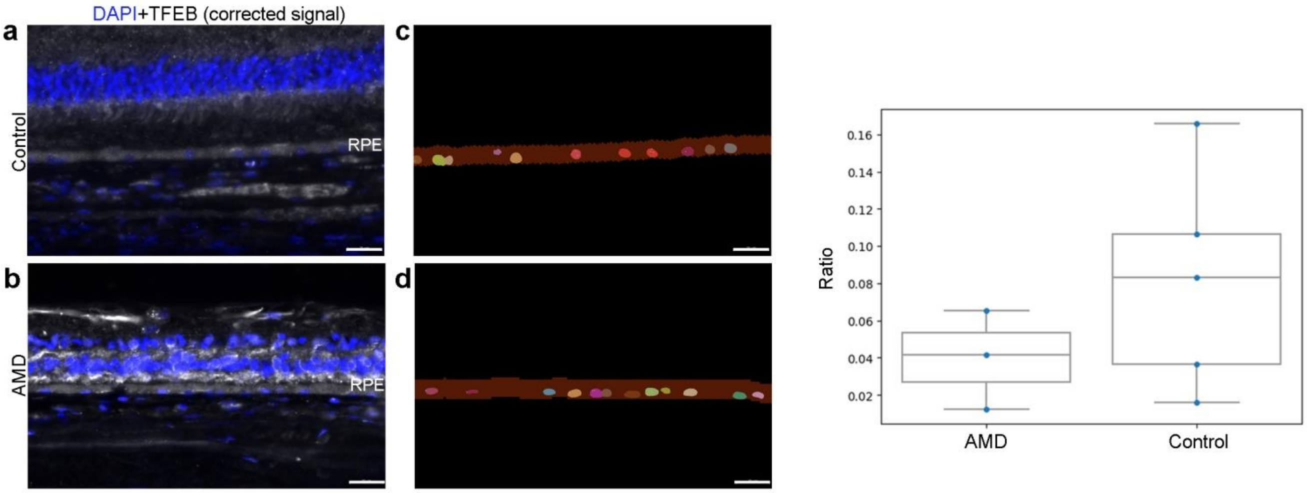

<u>Supplementary Figure 6</u>: ***TFEB expression in human AMD***. Immunohistochemistry followed by AI-based quantification on human donor retinal sections showing a trend towards decrease in the presence of TFEB nuclear foci (white) in (**b**) AMD patients, compared to (**a**) controls. (**c**, **d**) AI-based detection of RPE layer (brown), individual positive nuclei (multicolors). Nuclei were considered positive when containing at least two TFEB foci. Ratio of positive over all detected nuclei is presented in the boxplot. Scale bar= 25 μm. Control (n=5) and AMD (n=3). Source Data is provided in the Source Data file.

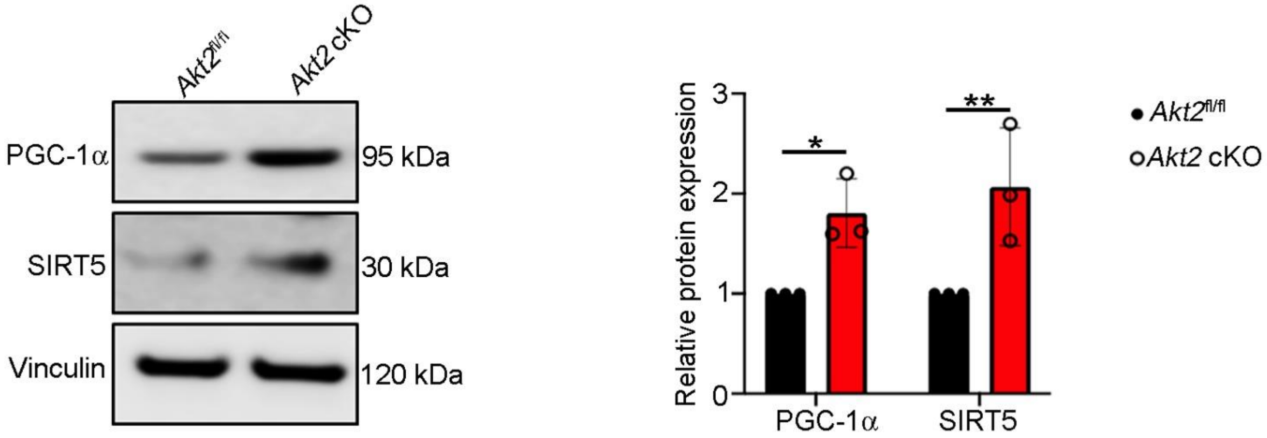

<u>Supplementary Figure 7</u>: ***Akt2 cKO RPE cells show upregulation in PGC-1α and SIRT5 levels***. Western blot showing elevated levels of both PGC-1α and SIRT5 in *Akt2* cKO RPE cells, relative to floxed controls (*Akt2*$^{fl/fl}$). n=3. All values are Mean ± S.D. **P<0.01, *P<0.05. The statistical test used is Student's t-test. The exact p-values are PGC-1α: P=0.0398 and SIRT5: P=0.0098 (*Akt2* cKO vs *Akt2*$^{fl/fl}$). Source Data is provided in the Source Data file.

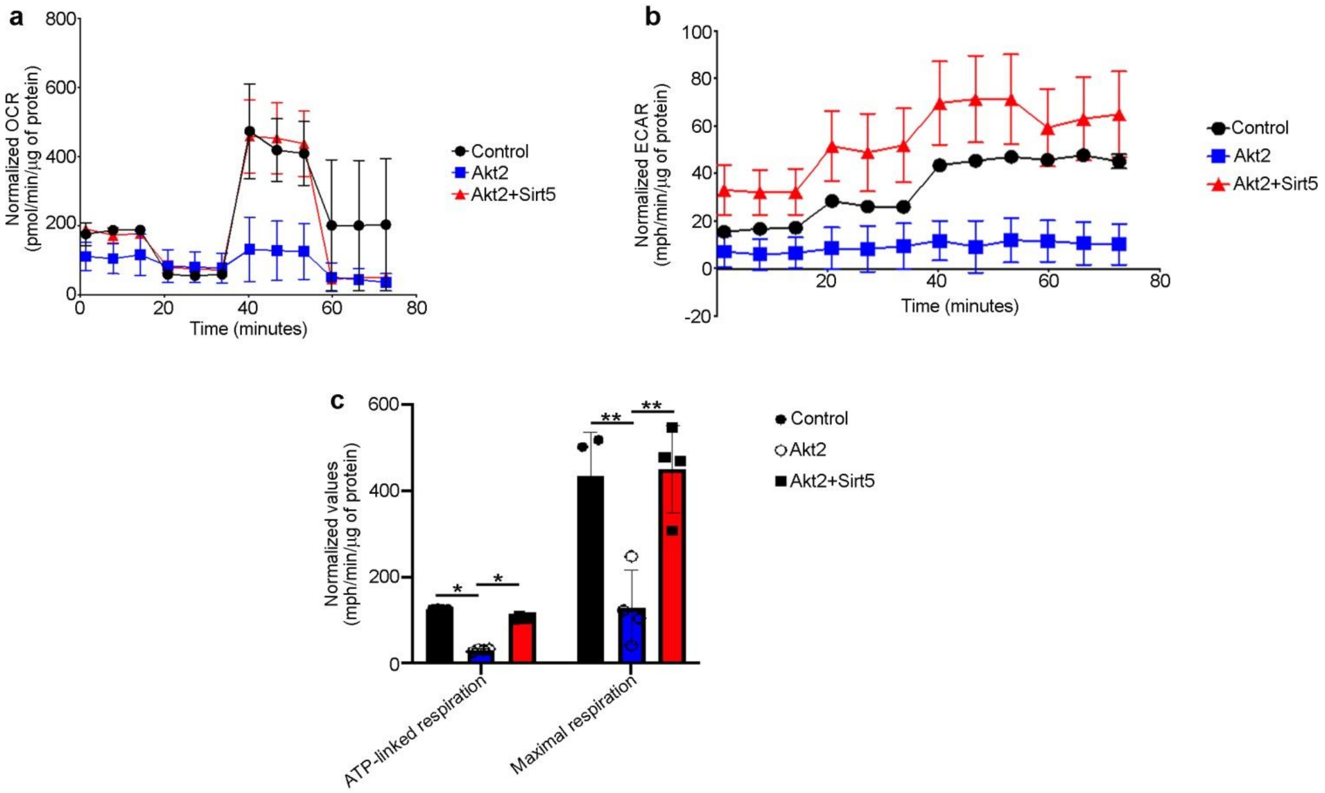

<u>Supplementary Figure 8</u>: ***Alterations in mitochondrial function in RPE cells overexpressing AKT2***. Seahorse analysis using the mitostress assay showing time-dependent changes in metabolic flux. Plots show (**a**) oxygen consumption rate (OCR) and (**b**) extracellular acidification rate (ECAR) upon treatment with mitochondrial respiration blockers oligomycin, Carbonyl cyanide-p-trifluoromethoxy phenylhydrazone (FCCP), and Rotenone/Antimycin A at particular time points in untransfected ARPE19 cells (control) or cells overexpressing AKT2 or cells overexpressing both AKT2 and SIRT5. n=4. (**c**) ATP-linked respiration and maximal respiration were significantly decreased in ARPE19 cells overexpressing AKT2, compared to controls. Such changes were rescued in cells overexpressing both AKT2 and SIRT5, indicating that SIRT5 can inhibit AKT2-mediated mitochondrial alterations. n=4. All values are Mean ± S.D. **P<0.01, *P<0.05. The statistical test used in (**c**) One-way ANOVA followed by Tukey’s post-hoc test. The exact p-values are for ATP-linked respiration: P=0.037 (Akt2 vs control) and P=0.045 (Akt2+Sirt5 vs Akt2); Maximal

respiration: P=0.0059 (Akt2 vs control) and P=0.0082 (Akt2+Sirt5 vs Akt2). Source Data is provided in the Source Data file.

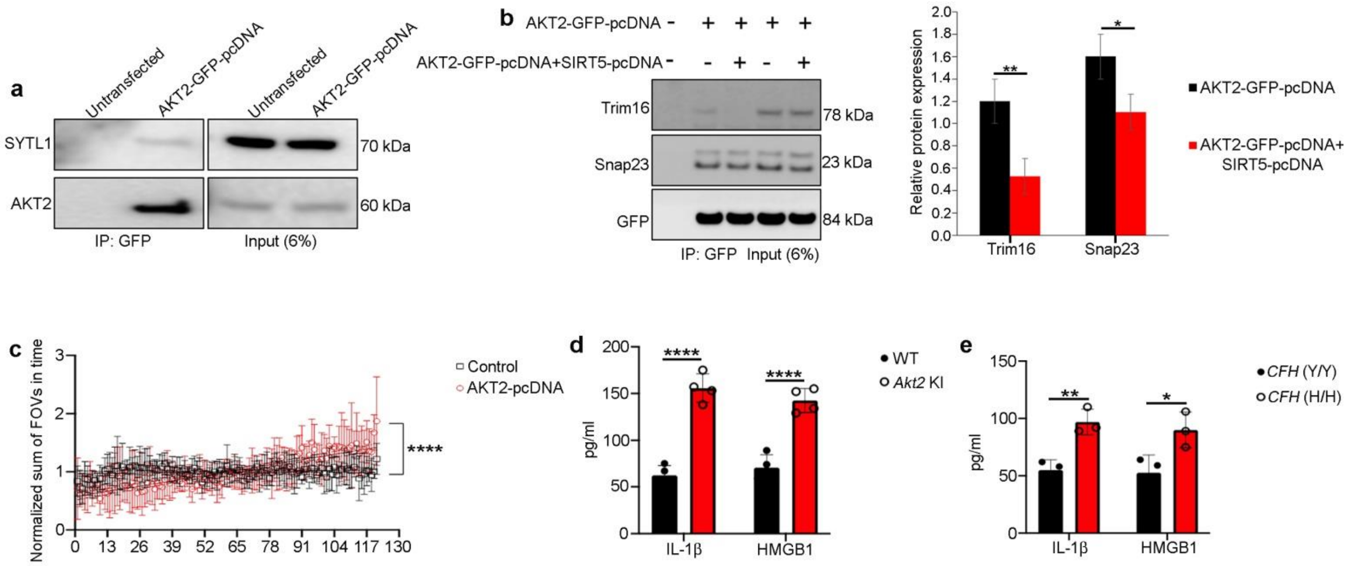

<u>Supplementary Figure 9</u>: ***Akt2 upregulation is associated with activation of secretory autophagy in the RPE cells.*** (**a**) Co-immunoprecipitation studies showing binding of SYTL1 to AKT2 in ARPE19 cells overexpressing AKT2-GFP when AKT2 was pulled down using anti-GFP magnetic beads. n=3. (**b**) Pulldown assay with anti-GFP magnetic beads from lysates of ARPE19 cells overexpressing either GFP-AKT2 or GFP-AKT2 and SIRT5-HA and serum/nutrient starved for 1h in HBSS. Cells overexpressing only GFP-AKT2 showed an increase in binding of secretory autophagy mediators Snap23 and Trim16 compared to untransfected cells. This binding was reduced upon simultaneous overexpression of SIRT5. n=4. (**c**) Linear regression plot showing significant change in autophagosome number on the cell membrane as evident from TIRF microscopy in AKT2 overexpressing (Akt2-pcDNA transfected) ARPE19 cells, compared to untransfected controls (X-axis= Normalized FOV; field of view), Y-axis= time (in minutes). n=3. ELISA showing increased levels of IL-1β and HMGB1 in spent medium from cultured (**d**) *Akt2* KI RPE explants and (**e**) iPSC-derived RPE cells from *CFH* Y402H risk allele containing donors [*CFH* (H/H)], compared to controls. n=3. All values are Mean ± S.D. ****P<0.0001, **P<0.01, *P<0.05. The statistical test used in (**b**) One-way

ANOVA followed by Tukey's post-hoc test, (**c**) simple linear regression, (**d**,**e**) Student's t-test. The exact p-values are (**b**) Trim16: P=0.038 and Snap23: P=0.041 (AKT2-GFP-pcDNA+SIRT5-pcDNA vs AKT2-GFP-pcDNA); (**c**) P= 0.000088 (AKT2-pcDNA vs control); (**d**) IL-1β: P=0.000047, HMGB1: P=0.000052 (*Akt2* KI vs WT); (**e**) IL-1β: P=0.0089, HMGB1: P=0.0166 (CFH(H/H) vs CFH (Y/Y)). Source Data is provided in the Source Data file.

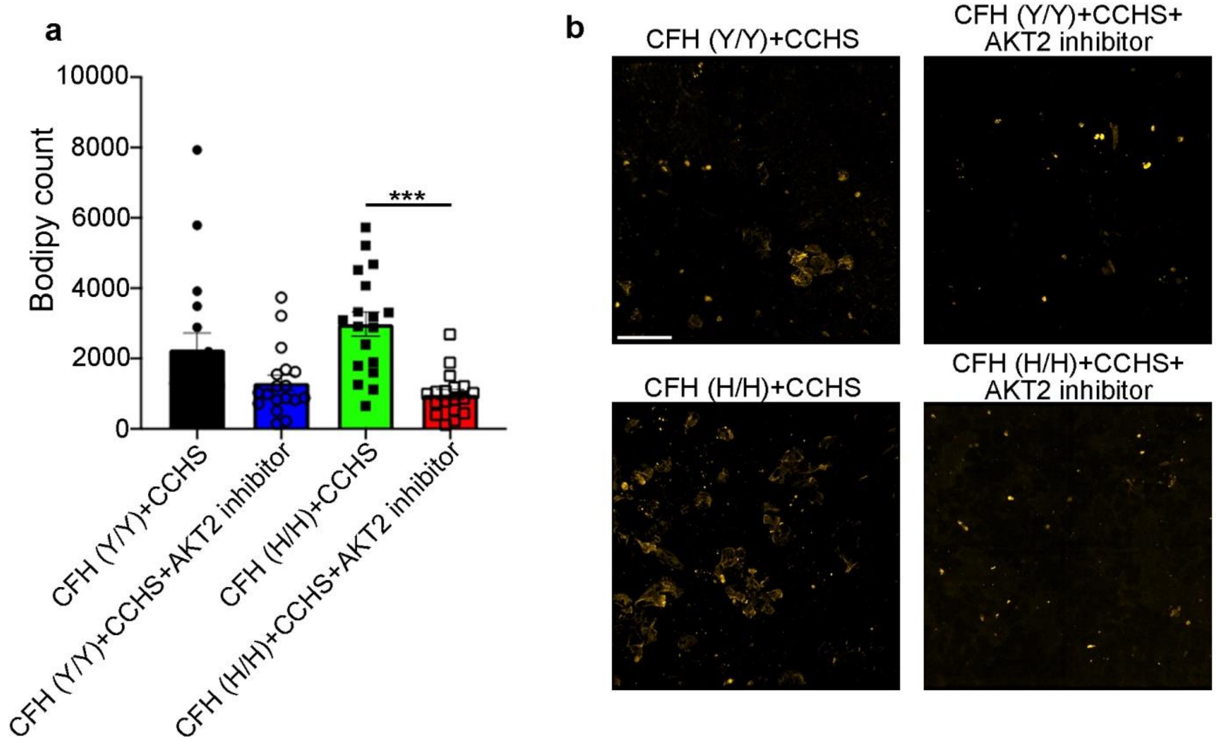

<u>Supplementary Figure 10</u>: AKT2 inhibitor treatment could rescue some salient features of in vitro AMD-like model. (**a**) AKT2 inhibitor (10 nM for 48 h) treatment to CFH (H/H) cells exposed to CCHS rescued lipid accumulation, as observed by decrease in Bodipy count compared to untreated CFH (H/H)+ CCHS cells. (**b**) Immunofluorescence staining with APOE antibody (yellow) showed a possible decreasing trend of this apolipoprotein accumulation upon AKT2 inhibitor treatment in both CCHS-exposed CFH (Y/Y) and (H/H) cells. n=3. Scale= 50 μm. ***P<0.001. The statistical test used in (**a**) One-way ANOVA followed by Tukey's post-hoc test. The exact p-values are (**a**) P=0.0002 (CFH (H/H)+CCHS+AKT2 inhibitor vs CFH (H/H)+CCHS) Source Data is provided in the Source Data file.

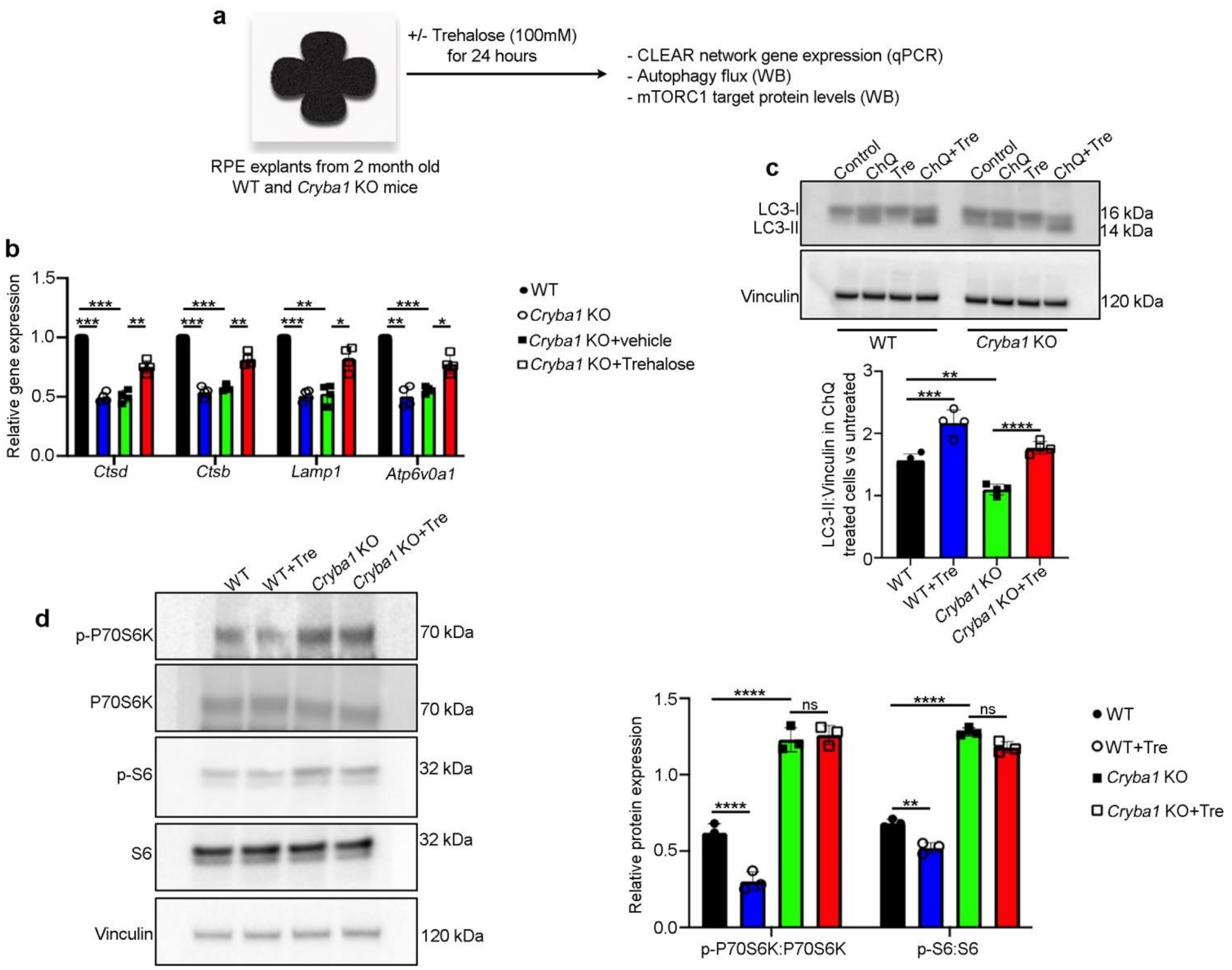

<u>Supplementary Figure 11</u>: ***Trehalose rescues lysosomal and autophagy abnormalities in Cryba1 KO RPE explants***. (**a**) Schematic showing experimental design to evaluate efficacy of trehalose in rescuing autophagy and lysosomal abnormalities in *Cryba1* KO RPE explants. (**b**) qPCR showing trehalose (100 mM for 24 h) treatment of *Cryba1* KO RPE explants could rescue the expression levels of CLEAR network genes like *Ctsd*, *Ctsb*, *Lamp1* and *Atp6v0a1*. n=4. (**c**) Western blot showing trehalose treatment could rescue the decrease in autophagy flux (Ratio of LC3-II/ Vinculin in ChQ treated vs untreated) in *Cryba1* KO RPE explants when treated with chloroquine (ChQ; 50 μm) for 6 h, compared to untreated *Cryba1* KO RPE explants. n=3. (**d**) Western blot showing no noticeable difference in mTORC1 downstream

mediators P70S6K and S6 in *Cryba1* KO RPE explants treated with trehalose, indicating that the effect of trehalose is independent of mTORC1 signaling. n=3. All values are Mean ± S.D. ****P<0.0001, ***P<0.001, **P<0.01, *P<0.05. ns=not significant. The statistical analysis used in (**b-d**) One-way ANOVA followed by Tukey's post-hoc test. The exact p-values are (**b**) *Ctsd*: P=0005 (*Cryba1* KO vs WT), P=0010 (*Cryba1* KO+ vehicle vs WT), P=0.002 (*Cryba1* KO+ Trehalose vs *Cryba1* KO+vehicle), *Ctsb*: P=0009 (*Cryba1* KO vs WT), P=0002 (*Cryba1* KO+ vehicle vs WT), P=0.0034 (*Cryba1* KO+ Trehalose vs *Cryba1* KO+vehicle), *Lamp1*: P=0008 (*Cryba1* KO vs WT), P=0043 (*Cryba1* KO+ vehicle vs WT), P=0.0172 (*Cryba1* KO+ Trehalose vs *Cryba1* KO+vehicle), *Atp6v0a1*: P=0041 (*Cryba1* KO vs WT), P=0002 (*Cryba1* KO+ vehicle vs WT), P=0.0244 (*Cryba1* KO+ Trehalose vs *Cryba1* KO+vehicle); (**c**) P=0.0002 (WT+Tre vs WT), P=0.0016 (*Cryba1* KO vs WT), P=0.000081 (*Cryba1* KO+Tre vs *Cryba1* KO); (**d**) p-P70S6K: P70S6K; P=0.000067 (WT+Tre vs WT), P=0.000055 (*Cryba1* KO vs WT), P=0.8930(*Cryba1* KO+Tre vs *Cryba1* KO), p-S6: S6; p-P70S6K: P70S6K; P=0.0070 (WT+Tre vs WT), P=0.000072 (*Cryba1* KO vs WT), P=0.1268 (*Cryba1* KO+Tre vs *Cryba1* KO). Source Data is provided in the Source Data file.

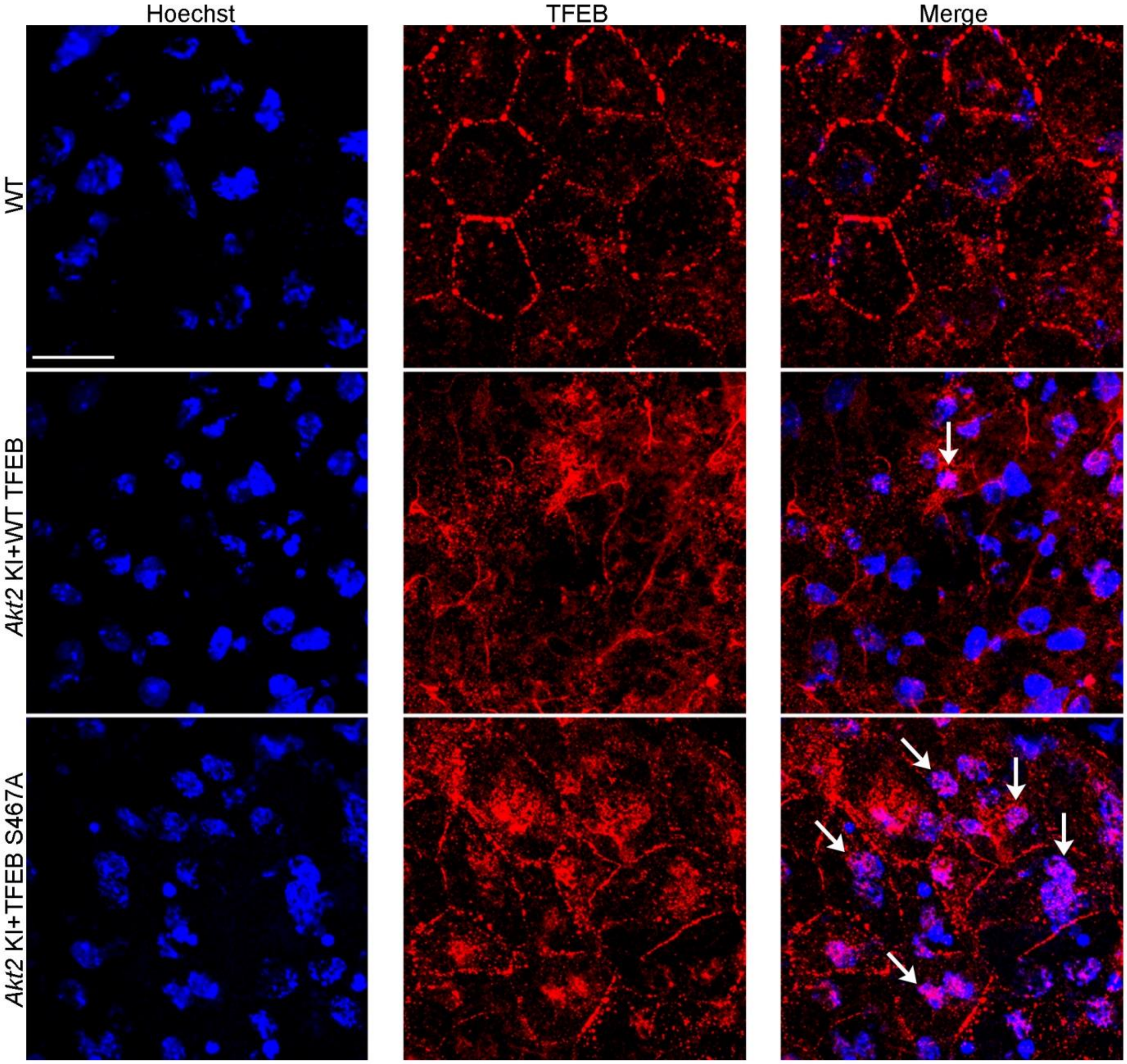

<u>Supplementary Figure 12</u>: TFEB nuclear translocation in RPE cells upon AAV2-TFEB-S467A infection. RPE explants from 4 month old WT and *Akt2* KI mice were cultured, followed by infection with AAV2-WT-TFEB or AAV2-TFEB-S467A constructs to *Akt2* KI explants for 24 h, followed by TFEB (Red) immunostaining (counterstained by DAPI; Blue) which showed significant increase in nuclear localization (arrows; magenta) in AAV2-TFEB-S467A infected explants, compared to WT-TFEB construct, though both constructs could successfully overexpress TFEB in the cytosol, when compared to WT. This indicates that

TFEB-S467A is essential for constitutive nuclear localization of the transcription factor in RPE cells. n=4. Scale= 20 μm.

**iPSC CELL LINE**	**GENOTYPE**	CFH (H/H) homo
D3C	CFH Y402H (H/H)	iRPE5
D2C		iRPE6
LORCF1-Z8		iRPE7
		CFH (Y/Y) wild type
BEST4C	CFH Y402 (Y/Y)	iRPE1
D4A		iRPE2
D4C		iRPE3

<u>Supplementary Table 1</u>. iPSC-cell lines used in the study from donors with no disease.

Name	ID	Z Score	S Score	F635	B635
SIRT5	JHU04487.B1C25R68	66.32	34.394	1576.5	47
SORBS3	JHU04395.B1C9R68	31.926	9.929	788.5	48.5
MAB21L1	JHU04362.B2C17R68	21.997	1.244	561	48
HIST1H1A	JHU10506.B8C29R78	20.753	4.147	532.5	48.5
H1FX	JHU08674.B6C11R48	16.606	0.087	437.5	49.5
PPP1R3B	JHU05921.B5C14R2	16.519	0.022	435.5	46.5
SCL-70	Auto-antigen.B20C6R42	16.497	0.109	435	49
CXorf51B	JHU10867.B5C14R84	16.388	0.589	432.5	48
CYB561	JHU13134.B9C27R28	15.799	0.000	419	48
SYTL1	JHU08341.B8C4R38	15.799	1.332	419	47.5
DIMT1	JHU09043.B8C6R54	14.467	0.392	388.5	47.5
PNKP	JHU08225.B5C9R42	14.075	0.284	379.5	47
C7orf50	JHU08406.B8C21R38	13.791	0.066	373	48
THYN1	JHU16333.B10C4R76	13.725	0.000	371.5	48.5
JHU04032	JHU04032.B3C6R62	13.725	0.589	371.5	48.5
Lupus La	Auto-antigen.B20C4R38	13.136	0.458	358	49
SRSF5	JHU04389.B9C3R88	12.678	0.044	347.5	54.5
VRK1	JHU10925.B7C27R80	12.634	0.218	346.5	48
ANXA2	JHU13600.B12C25R36	12.416	0.502	341.5	48.5
KHDRBS1	JHU15238.B9C18R60	11.914	0.065	330	47
GYS1	JHU08863.B5C3R52	11.849	0.022	328.5	48

Supplementary Table 2: ***AKT2 binding partners***. Human high-throughput protein-protein interaction study showing several AKT2 binding partners with their Z-scores. SIRT5 and SYTL1 are highlighted with red and blue, respectively.

iPSC-RPE cell line	CFH genotype	Risk
LR 1	TT	Low Risk (LR)
LR 2	TT	
LR 3	TT	
HR 1	CC	High Risk (HR)
HR 2	CC	
HR 3	CC	
HR 4	CC	

Supplementary Table 3: iPSC-cell lines used in the study from conjunctiva of donors with AMD.
