# Supplementary material for: The AKT2/SIRT5/TFEB pathway as a potential therapeutic target in non-neovascular AMD
Source: Nat Commun. 2024 Jul 21;15:6150. doi: 10.1038/s41467-024-50500-z (PMC11271488; doi:10.1038/s41467-024-50500-z)
Supplement: Supplementary file 3 — Description of Additional Supplementary Files [file 41467_2024_50500_MOESM3_ESM.pdf]

Description of Additional Supplementary Materials:

Supplementary Movie 1: ***TIRF imaging for autophagosome binding to cell surface***. Time lapse movie from control ARPE19 cells showing GFP-LC3 puncta at the cell surface over as a function pf time, upon induction of starvation (incubation in serum free medium). n=3.

Supplementary Movie 2: TIRF imaging for autophagosome binding to cell surface. Time lapse movie from Akt2 overexpressing ARPE19 cells showing GFP-LC3 puncta at the cell surface over as a function of time, upon induction of starvation (incubation in serum free medium). n=3.
