# Supplementary material for: The AKT2/SIRT5/TFEB pathway as a potential therapeutic target in non-neovascular AMD
Source: Nat Commun. 2024 Jul 21;15:6150. doi: 10.1038/s41467-024-50500-z (PMC11271488; doi:10.1038/s41467-024-50500-z)
Supplement: Supplementary file 6 — Reporting Summary [file 41467_2024_50500_MOESM6_ESM.pdf]

nature portfolio

Corresponding author(s): James T. Handa & Debasish Sinha

Last updated by author(s): June 21, 2024

Reporting Summary

Nature Portfolio wishes to improve the reproducibility of the work that we publish. This form provides structure for consistency and transparency in reporting. For further information on Nature Portfolio policies, see our Editorial Policies and the Editorial Policy Checklist.

Please do not complete any field with "not applicable" or n/a. Refer to the help text for what text to use if an item is not relevant to your study.
For final submission: please carefully check your responses for accuracy; you will not be able to make changes later.

Statistics

For all statistical analyses, confirm that the following items are present in the figure legend, table legend, main text, or Methods section.

n/a	Confirmed	
☐	☒	The exact sample size (*n*) for each experimental group/condition, given as a discrete number and unit of measurement
☐	☒	A statement on whether measurements were taken from distinct samples or whether the same sample was measured repeatedly
☐	☒	The statistical test(s) used AND whether they are one- or two-sided<br>*Only common tests should be described solely by name; describe more complex techniques in the Methods section.*
☒	☐	A description of all covariates tested
☐	☒	A description of any assumptions or corrections, such as tests of normality and adjustment for multiple comparisons
☐	☒	A full description of the statistical parameters including central tendency (e.g. means) or other basic estimates (e.g. regression coefficient) AND variation (e.g. standard deviation) or associated estimates of uncertainty (e.g. confidence intervals)
☐	☒	For null hypothesis testing, the test statistic (e.g. *F*, *t*, *r*) with confidence intervals, effect sizes, degrees of freedom and *P* value noted<br>*Give P values as exact values whenever suitable.*
☒	☐	For Bayesian analysis, information on the choice of priors and Markov chain Monte Carlo settings
☒	☐	For hierarchical and complex designs, identification of the appropriate level for tests and full reporting of outcomes
☒	☐	Estimates of effect sizes (e.g. Cohen's *d*, Pearson's *r*), indicating how they were calculated

Our web collection on statistics for biologists contains articles on many of the points above.

Software and code

Policy information about availability of computer code

Data collection	Zen software (Zeiss) version 2.3, Azure Biosystems c200 imaging system (Azure Biosystems), Celeris Diognosys System, Proteome Discoverer (PD 2.5, ThermoFisher Scientific), Cellpose, NIS ELements
Data analysis	Excel (Microsoft), GraphPad Prism v9, Mascot (version 2.8.0), Scaffold (version Scaffold_5.0.1), ABI Biosystems, NIS Elements spot detection software, ImageJ (National Institute of Health)

For manuscripts utilizing custom algorithms or software that are central to the research but not yet described in published literature, software must be made available to editors and reviewers. We strongly encourage code deposition in a community repository (e.g. GitHub). See the Nature Portfolio guidelines for submitting code & software for further information.

Data

Policy information about availability of data

All manuscripts must include a data availability statement. This statement should provide the following information, where applicable:

- Accession codes, unique identifiers, or web links for publicly available datasets
- A description of any restrictions on data availability
- For clinical datasets or third party data, please ensure that the statement adheres to our policy

All data generated or analysed during this study are included in this published article (and its Supplementary Information files). The RNAseq data for the genes depicted in Fig. 4c has been uploaded in the NCBI GEO database (Accession number: GSE269923). All rights and ownership of BioRender content are reserved by BioRender. All completed graphics must be accompanied by the following citation: “Created with BioRender.com”.

Research involving human participants, their data, or biological material

Policy information about studies with human participants or human data. See also policy information about sex, gender (identity/presentation), and sexual orientation and race, ethnicity and racism.

Reporting on sex and gender	
Reporting on race, ethnicity, or other socially relevant groupings	
Population characteristics	
Recruitment	
Ethics oversight	Human cadaver donor paraffin embedded eye globes were commercially purchased from Lion's Gift of Sight, Minnesota eye bank. The ethical approvals and protocol maintenance is by LIon's Gift of Sight

Note that full information on the approval of the study protocol must also be provided in the manuscript.

Field-specific reporting

Please select the one below that is the best fit for your research. If you are not sure, read the appropriate sections before making your selection.

☐ Life sciences ☐ Behavioural & social sciences ☐ Ecological, evolutionary & environmental sciences

For a reference copy of the document with all sections, see nature.com/documents/nr-reporting-summary-flat.pdf

Life sciences study design

All studies must disclose on these points even when the disclosure is negative.

Sample size	Sample sizes were determined based on power calculation, showing a difference of 25% and up to 20% SD, alpha=0.05 and beta=0.2
Data exclusions	No data were excluded, but some samples (<1%) were excluded from the experiments due to technical challenges during tissue collection
Replication	At least 3 biological replicates were used to reliably reproduce the results of the experiments.
Randomization	Animals (from the respective genotypes) were randomly allocated to each group
Blinding	To eliminate bias, individuals handing core-facility instruments or performing analysis on specific experiments were blinded to mouse genotype identity as well as the identity of the experimental groups.

Behavioural & social sciences study design

All studies must disclose on these points even when the disclosure is negative.

Study description	
Research sample	
Sampling strategy	
Data collection	
Timing	
Data exclusions	
Non-participation	
Randomization	

Ecological, evolutionary & environmental sciences study design

All studies must disclose on these points even when the disclosure is negative.

Study description	
Research sample	
Sampling strategy	
Data collection	
Timing and spatial scale	
Data exclusions	
Reproducibility	
Randomization	
Blinding	

Did the study involve field work? ☐ Yes ☐ No

Field work, collection and transport

Field conditions	
Location	
Access & import/export	
Disturbance	

Reporting for specific materials, systems and methods

We require information from authors about some types of materials, experimental systems and methods used in many studies. Here, indicate whether each material, system or method listed is relevant to your study. If you are not sure if a list item applies to your research, read the appropriate section before selecting a response.

Materials & experimental systems

n/a	Involved in the study	
☐	☒	Antibodies
☐	☒	Eukaryotic cell lines
☒	☐	Palaeontology and archaeology
☐	☒	Animals and other organisms
☒	☐	Clinical data
☒	☐	Dual use research of concern
☒	☐	Plants

Methods

n/a	Involved in the study	
☒	☐	ChIP-seq
☒	☐	Flow cytometry
☒	☐	MRI-based neuroimaging

Antibodies

Antibodies used	The primary antibodies AKT2 (3063S), p-AKT2 (8599S), SIRT5 (8782S), CTSD (69854S), ULK1 (8054T), FKBP51 (8245S), p-TFEB (37681S), and GFP (2555S) were purchased from Cell Signaling Technology. CTSL (NB100-1775), p62/SQSTM1 (NBP1-42821) and PGC-1a (NBP1-04676) were purchased from Novus Biologicals. TFE3 (14480-1-AP), ATG9B (PA5-20998), Ezrin (PA518541), Trim16 (PA5-110515), and Snap23 (PA1-738) were purchased from Thermo Fisher. Loading controls were vinculin (ab129002, Abcam) and beta-Actin (4970S, Cell Signaling). TFEB (A303-673A) and SYTL1 (A305-648A-T) antibodies were purchased from Bethyl Laboratories. The p-TFE3 antibody was a gift from Dr. Rosa Puertollano's laboratory. The secondary antibodies used in this study were purchased from KPL: anti-rabbit (074–1506) and anti-mouse (074–1806).
Validation	The validation information of the antibodies used in this study is provided in the manufacturers website.

Eukaryotic cell lines

Policy information about cell lines and Sex and Gender in Research

Cell line source(s)	ARPE19 cells (ATCC; CRL2302, Human iPSC-derived RPE cell line and iRPE (CFH null) cells (From Kapil Bharti lab at NEI, National Institute of Health
Authentication	iPSCs and iRPE: Culturing and authentication was performed following previous publications as mentioned by the text; ARPE19: ATCC datasheet for validation was obtaned
Mycoplasma contamination	No mycoplasma contamination was found during human fetal RPE culture
Commonly misidentified lines (See ICLAC register)	N/A

Palaeontology and Archaeology

Specimen provenance	
Specimen deposition	
Dating methods	

☐ Tick this box to confirm that the raw and calibrated dates are available in the paper or in Supplementary Information.

Ethics oversight	

Note that full information on the approval of the study protocol must also be provided in the manuscript.

Animals and other research organisms

Policy information about studies involving animals; ARRIVE guidelines recommended for reporting animal research, and Sex and Gender in Research

Laboratory animals	RPE specific Akt2 KI mice, Akt2 cKO, Akt2-floxed, wild type (WT), Cryba1-floxed and Cryba1 cKO male and female mice were used in this study and the animals were in the age range of 1-15months. Housing was overseen by the DLAR, UPMC Children's Hospital in individually-ventilated polysulfone rodent cages, supplied ventilation via a HEPA-filtered supply, wall-mounted unit.Exhaust is connected to the building's exhaust fans with a flexible hose assembly. The temperature and humidity are controlled and there is a 12 hour light and dark cycle.
Wild animals	N/A
Reporting on sex	Animals of both sexes were used in the study but no statistical difference in any of the parameters tested were observed between the two sexes
Field-collected samples	N/A
Ethics oversight	All animal studies were conducted in accordance with the guide for the care and use of animals (National Academy Press) and were approved by the Institutional Animal Care and Use Committee of the University of Pittsburgh. Protocol number is 20108281.

Note that full information on the approval of the study protocol must also be provided in the manuscript.

Clinical data

Policy information about clinical studies

All manuscripts should comply with the ICMJE guidelines for publication of clinical research and a completed CONSORT checklist must be included with all submissions.

Clinical trial registration	
Study protocol	
Data collection	
Outcomes	

Dual use research of concern

Policy information about dual use research of concern

Hazards

Could the accidental, deliberate or reckless misuse of agents or technologies generated in the work, or the application of information presented in the manuscript, pose a threat to:

No	Yes	
X	☐	Public health
X	☐	National security
X	☐	Crops and/or livestock
X	☐	Ecosystems
X	☐	Any other significant area

Experiments of concern

Does the work involve any of these experiments of concern:

No	Yes	
X	☐	Demonstrate how to render a vaccine ineffective
X	☐	Confer resistance to therapeutically useful antibiotics or antiviral agents
X	☐	Enhance the virulence of a pathogen or render a nonpathogen virulent
X	☐	Increase transmissibility of a pathogen
X	☐	Alter the host range of a pathogen
X	☐	Enable evasion of diagnostic/detection modalities
X	☐	Enable the weaponization of a biological agent or toxin
X	☐	Any other potentially harmful combination of experiments and agents

Plants

Seed stocks	
Novel plant genotypes	
Authentication	

ChIP-seq

Data deposition

☐ Confirm that both raw and final processed data have been deposited in a public database such as GEO.

☐ Confirm that you have deposited or provided access to graph files (e.g. BED files) for the called peaks.

Data access links<br>*May remain private before publication.*	
Files in database submission	
Genome browser session<br>(e.g. UCSC)	

Methodology

Replicates	
Sequencing depth	
Antibodies	
Peak calling parameters	
Data quality	
Software	

Flow Cytometry

Plots

Confirm that:

☐ The axis labels state the marker and fluorochrome used (e.g. CD4-FITC).

☐ The axis scales are clearly visible. Include numbers along axes only for bottom left plot of group (a 'group' is an analysis of identical markers).

☐ All plots are contour plots with outliers or pseudocolor plots.

☐ A numerical value for number of cells or percentage (with statistics) is provided.

Methodology

Sample preparation

Instrument

Software

Cell population abundance

Gating strategy

☐ Tick this box to confirm that a figure exemplifying the gating strategy is provided in the Supplementary Information.

Magnetic resonance imaging

Experimental design

Design type

Design specifications

Behavioral performance measures

Imaging type(s)

Field strength

Sequence & imaging parameters

Area of acquisition

Diffusion MRI ☐ Used ☐ Not used

Preprocessing

Preprocessing software

Normalization

Normalization template

Noise and artifact removal

Volume censoring

Statistical modeling & inference

Model type and settings

Effect(s) tested

Specify type of analysis: ☐ Whole brain ☐ ROI-based ☐ Both

Statistic type for inference

(See Eklund et al. 2016)

Correction

Models & analysis

n/a	Involved in the study
☐	☐ Functional and/or effective connectivity
☐	☐ Graph analysis
☐	☐ Multivariate modeling or predictive analysis

Functional and/or effective connectivity

Graph analysis

Multivariate modeling and predictive analysis

This checklist template is licensed under a Creative Commons Attribution 4.0 International License, which permits use, sharing, adaptation, distribution and reproduction in any medium or format, as long as you give appropriate credit to the original author(s) and the source, provide a link to the Creative Commons license, and indicate if changes were made. The images or other third party material in this article are included in the article's Creative Commons license, unless indicated otherwise in a credit line to the material. If material is not included in the article's Creative Commons license and your intended use is not permitted by statutory regulation or exceeds the permitted use, you will need to obtain permission directly from the copyright holder. To view a copy of this license, visit http://creativecommons.org/licenses/by/4.0/
